# Ostracism modulates children’s recognition of emotional facial expressions

**DOI:** 10.1371/journal.pone.0287106

**Published:** 2023-06-15

**Authors:** Julia Mermier, Ermanno Quadrelli, Hermann Bulf, Chiara Turati

**Affiliations:** 1 Department of Psychology, University of Milano-Bicocca, Milan, Italy; 2 NeuroMI, Milan Center for Neuroscience, Milan, Italy; The University of Faisalabad, PAKISTAN

## Abstract

Ostracism has been shown to induce considerable physiological, behavioral and cognitive changes in adults. Previous research demonstrated its effects on children’s cognitive and behavioral abilities, but less is known about its impact on their capacity to recognize subtle variations in social cues. The present study aimed at investigating whether social manipulations of inclusion and ostracism modulate emotion recognition abilities in children, and whether this modulation varies across childhood. To do so, 5- and 10-year-old children participated in a computer-based ball tossing game called Cyberball during which they were either included or ostracized. Then, they completed a facial emotion recognition task in which they were required to identify neutral facial expressions, or varying levels of intensity of angry and fearful facial expressions. Results indicated lower misidentification rates for children who were previously ostracized as compared to children who were previously included, both at 5 and 10 years of age. Moreover, when looking at children’s accuracy and sensitivity to facial expressions, 5-year-olds’ decoding abilities were affected by the social manipulation, while no difference between included and ostracized participants was observed for 10-year-olds. In particular, included and ostracized 10-year-old children as well as ostracized 5-year-olds showed higher accuracy and sensitivity for expressions of fear as compared to anger, while no such difference was observed for included 5-year-olds. Overall, the current study presents evidence that Cyberball-induced inclusion and ostracism modulate children’s recognition of emotional faces.

## Introduction

Human beings heavily rely on social interactions to survive and thrive in their environment [1, 2]. All forms of social exclusion, such as ostracism (i.e., being ignored and excluded) or rejection (i.e., being explicitly informed that one is not wanted), threaten primary needs such as belonging, control, self-esteem and sense of meaningful existence in adults [3, for a review see 4], as well as in children [5].

According to Williams [6], ostracism can lead either to prosocial or antisocial behaviours, depending on the likelihood of being re-included in the group and on the psychological need that is most threatened. If re-inclusion is perceived as likely, belonging and self-esteem needs will trigger prosocial behaviours, aiming at fortifying relationships and being re-accepted in the group. For instance, participants demonstrated to be more compliant to others’ requests and mimicked others’ facial emotions more after being ostracized than after being included in an online ball-tossing game (i.e., Cyberball) [7–12]. On the contrary, if re-inclusion is perceived as unlikely, the response will be driven by the need for control and meaningful existence, which will elicit anti-social behaviours meant to re-establish a sense of control for the individual, rather than facilitate its re-inclusion [4]. For example, ostracized participants displayed more aggressive behaviours (i.e., allocated more hot sauce or played aversive noise to a stranger) when experiencing a loss of control as compared to included participants and ostracized participants with higher levels of control [13, 14].

In addition to influencing behaviour, ostracism also induces noticeable attentional and cognitive changes. According to Gardner and colleagues [15], individuals possess a social monitoring system which, when detecting threats to their belonging needs, directs attentional and cognitive resources towards social cues, which in many cases are subtly expressed, as in the case of facial emotional expressions or vocal tones. In this way, they are better equipped to selectively detect socially relevant information that will subsequently facilitate the re-establishment of social connections and their re-inclusion. Numerous studies using different forms of social exclusion (e.g., rejection messages, ostracism via an online ball-tossing game or priming videos depicting third-party ostracism) provide evidence supporting the social monitoring model. For instance, excluded adults allocated more attention and were more accurate in identifying emotional vocal tones than included ones [16]. The social monitoring system has been proposed to be highly adaptive, as it is triggered when an individual’s current level of social inclusion is lower than their desired level. However, the allocation of attentional and cognitive resources towards socially relevant cues appears to be detrimental for non-social cognitive tasks, as demonstrated by the decrease in general cognitive performance (e.g., effortful logic and reasoning) in adults after experiencing social exclusion [17].

Available literature emphasizes the importance of group membership and the role of social cues in forming and maintaining social connections. Faces, being highly informative social cues, hold substantial value in this sense [18, 19]. Accurately interpreting facial expressions enables individuals to understand others’ emotions and internal dispositions, enhancing successful social interactions. Ostracism modulates emotion recognition abilities, leading to increased cognitive resources dedicated to emotion decoding for reconnection and re-inclusion. Research on adults showed that social ostracism improved the decoding of static and dynamic facial expressions of happiness, anger, fear, sadness and disgust [12, 16, 20]. In addition, excluded participants were better at categorizing angry versus happy facial expressions [21] and at discriminating “fake” from “real” smiles [22] than included ones.

A growing body of research suggests that ostracism induces considerable behavioural and cognitive changes also at the earliest stages of development [23]. For example, at 5 years of age, ostracized children displayed more affiliative behaviours such as increased imitation of others’ actions or language choices as compared to included ones [24, 25]. In addition, the effects of social ostracism are so robust that merely witnessing someone else being excluded prompted children to sit closer to a stranger [26], draw more affiliative pictures [27] and imitate more accurately others’ actions [28, 29]. Similarly to adults, and in line with the social monitoring model [15], ostracism also seemed to drive children’s attentional and cognitive resources towards social cues, while having detrimental effects on non-social tasks. For instance, 5-year-old children who witnessed third-party exclusion or were excluded themselves showed selective memory for social events and items [26, 30], while 8- to 12-year-old girls (but not boys) displayed lower cognitive performances on non-social tasks [31]. While available studies suggest that sensitivity to ostracism appears early in life [23], little is known about whether and how it affects children’s ability to recognize facial expressions of emotions.

The current study aimed at investigating whether social ostracism modulates emotion recognition abilities in 5- and 10-year-old children, thus exploring whether this modulation varies across preschool- and school-aged children. A substantial number of studies demonstrated that early in life humans not only detect regularities and learn from predictable sequences of emotional faces [32, 33], but also display an attentional bias and exhibit a complex neural network involved in the processing of facial emotional expressions [34–39]. However, substantial changes in the ability to recognize facial expressions of emotions occur between preschool and school ages. Indeed, an increase in facial recognition accuracy has been found between 3 and 7 years of age [40–42], with improvement in processing speed between 7 and 10 years of age [43] and in the ability to recognize subtle facial expressions of emotions between 5 and 10 years of age [44], to become adult-like only through the course of adolescence [45, 46]. Moreover, it has been shown that ostracism experiences between kindergarten and the 5th grade reduce children’s classroom participation, increase school avoidance and delay their school achievement [47], again suggesting that preschool and school years represent crucial time points to investigate children’s reaction to an ostracism experience. Capitalizing on evidence about the development of both emotion recognition processes and reactions to ostracism, our investigation is focused on 5- and 10-year-old children, which are also the extreme ages tested in the emotion recognition task used by Gao & Maurer [44] and employed in the current study.

Ostracism was induced using the well-established Cyberball paradigm [7], an online ball-tossing game in which children were either included (i.e., frequently received the ball during the entire game), or ostracized (i.e., received the ball only twice at the beginning of the game, and then never again). Already widely used in adults, recent research suggests that the Cyberball is equally efficient at inducing ostracism in children from 5 years of age [5, 28–31]. Following the Cyberball game, children participated in an emotion recognition task, adapted from the study by Gao & Maurer [44], in which they were asked to identify facial expressions of emotions displayed by women at different intensities. Given that our task comprised two different phases (i.e., the Cyberball paradigm and the facial emotion recognition task), in order to avoid a prolonged and tiring experimental session, we preferred to focus only on two facial expressions of emotions, specifically on two negative facial expressions (i.e., anger and fear). Indeed, positive facial expressions have been shown to be recognized much earlier and much more accurately than negative ones during development [e.g., 41, 42], thus they are likely less malleable to social exclusion manipulation. The emotions of fear and anger were chosen for their evolutionary function of signaling a potential danger, which might be relevant when exposed to a social threat such as ostracism. Moreover, fear and anger might be experienced when children are ostracized [6, 47–49]. We did not include sadness because data suggest that young children are more likely to misidentify it as neutral, disgusted, or fearful [50]. However, as in Gao and colleagues [44], we included neutral expressions to prevent children from thinking that all faces necessarily expressed a negative emotion.

Based on evidence of its robust effect in modulating individuals’ behaviour and cognition [3, 4, 15], we expected ostracism to induce considerable changes in children’s emotion recognition performance. In particular, consistent with previous adult studies [12, 16] and with Gardner and colleagues’ social monitoring model [3], we predicted that ostracized children would show heightened emotion recognition abilities as compared to included ones, anger and fear being especially relevant in socially-threatening situations such as ostracism. Alternatively, in agreement with most recent models on the development of emotion recognition abilities attributing distinct visual and neural processing in response to different emotions [37–39, 51, 52], it is also possible that the effects of ostracism may differ depending on the observed emotion. In line with the available literature, we expected age to modulate children’s ability to recognize emotions, with older children showing better emotion recognition performance than younger ones, but we presumed that in both age groups recognition of emotional expressions could be modulated by ostracism.

## Methods

### Participants

A total of 100 children was included in the final sample, from whom fifty 5-year-olds (28 girls; M_age_ = 5.38 years, SD_age_ = 0.31 years), and fifty 10-year-olds (25 girls; M_age_ = 10.49 years, SD_age_ = 0.22 years). In each age group, half of the participants was randomly assigned to the *inclusion* condition (N = 25), and the other half to the *ostracism* condition (N = 25). Five additional participants were tested but excluded from the final sample due to atypical motor development (N = 1 in the 5-year-old group), neurodevelopmental disorders (N = 1 in the 10-year-old group), because the child refused to take part in the task (N = 2 equally distributed in the two age groups), or because testing was not completed (N = 1 in the 5-year-old group). Participants were recruited from a local database of parents, who had previously volunteered to participate in child development studies. Children were recruited from a diverse urban environment including the metropolitan and suburban areas of Milano (Italy), characterized by approximately 75% of European individuals [53]. In order to participate in the study, participants had to be born with normal birth weight and no history of neurological disorders, or pre- or perinatal complications. All children had normal or corrected-to-normal vision. Based on the available literature using comparable procedures [47, 44, 50] and on an a priori power analysis conducted using the G*Power software [54], a sample size of 98 participants was estimated in order to have 80% probability to detect a significant interaction (α = .05) with a medium effect size (f = .25) in a mixed model, following Cohen’s guidelines [55]. Participants were recruited via written invitation based on birth records of neighboring cities. Informed written consent was given by both the parents and the children prior to the participation in the study. The procedure followed the ethical standards of the Declaration of Helsinki (BMJ 1991; 302: 1194) and was approved by the Ethical Committee of the University of Milano-Bicocca (Protocol number: 556).

### Procedure

The entire study took place online, so each child participated from home on a computer provided by her/his family. Once the parents accepted to participate in the study, families were sent a document via email containing a detailed description of the procedure, the instructions to complete the task, and a link to access it. Participants accessed the Qualtrics platform (Qualtrics, Provo, UT), where they were redirected to the Cyberball webpage. At this point, each child took part in a training phase to be briefly familiarized with the online catch-and-throw game and learn how to play [7]. Next, participants took part in the actual Cyberball phase, where the inclusion/ostracism manipulation occurred. Lastly, all children re-entered the Qualtrics platform, in which they were asked to identify the different emotions expressed on photographs of woman’s faces. Precise instructions were given to parents to sit back and never help the child, who had to complete the task by himself or herself. Parents’ intervention was allowed only when the child did not intend to continue the task; exclusively in this case, parents were instructed to encourage the child to continue the task. Once parents gave their consent for the participation of their child to the study, they passed on the computer to the child.

#### Cyberball paradigm

Before starting the Cyberball training phase, children were given detailed video instructions on how to play the game. They were told that they would play a ball tossing game with two other children, Laura and Marco, playing from home too. In reality, the other children were computer-programmed players, who were represented by a picture of a child’s face and an avatar shown at the bottom of the screen. After Williams and colleagues’ [7] well-established procedure, participants were instructed that when they had the ball, they could throw it to the player of their choice by clicking with the mouse on his or her image, and that when the other players had the ball, they could also decide who to throw it to. Finally, they were explained that the game was not a competition, but a game of imagination, and were asked to imagine being at the park, throwing the ball with Laura and Marco. The training phase consisted of a very brief version of the Cyberball. Children were told that this part was a training to learn how to play the game. The ball was placed in the hands of the avatar representing the child, who could decide to throw it to one of the other two avatars by clicking on it. The overall training phase lasted five throws, of which two were performed by the child.

Children were then instructed that the real game with the two other children was about to start. Children were randomly assigned to the social inclusion or ostracism condition. In both cases, the Cyberball game was programmed to last 18 throws (around 2 minutes overall). Children assigned to the *inclusion* condition received the ball a third of the time (i.e., 6 times out of a total of 18 throws), so that each player participated to the game equitably. Children assigned to the *ostracism* condition received the ball only twice at the beginning of the game and were then ignored by the other two players, who kept passing the ball to each other until they reached 18 throws.

The Cyberball paradigm was adapted to children and based on past research showing that its average effect was large and generalizable across structural (i.e., number of throws, duration of ostracism, etc.) and sampling (i.e., age, gender, nationality) aspects (for details, see the meta-analysis [3]). The number of throws was chosen based on Hawes and colleagues [31], who found the optimal number of 20 throws for children of 8 to 12 years of age, which was adjusted to 18 throws to make it equitable across players in case of inclusion (multiple of 3). Although most previous studies investigated the effects of Cyberball on adults, a few recent studies confirmed the robustness of the ostracism manipulation on children ranging from 5 to 10 years of age [5, 28–31]. On a qualitative account, parents of excluded children frequently (35% of 5-year-old children; 87% of 10-year-old children) reported that their child verbalized feelings of distress and sadness (e.g., “why did they stop passing me the ball?”, “I don’t like this game!”, or “I am not playing anymore”), while none of these signs was noted by the parents of included participants.

#### Emotion recognition task

Immediately after the Cyberball game, children participated in the emotion recognition task where they were asked to identify different expressions on photographs of woman faces (i.e., angry, fearful and neutral expressions). They were first informed that they would see photographs of either Flavia or Valentina, who sometimes felt angry, sometimes scared, and sometimes did not feel anything. Then, the experimenter asked the child to help him understand how the two friends felt. The photographs were presented one by one at the center of the screen, and below were displayed the three potential answers in the form of schematic faces posing angry, fearful or neutral expressions. Children were asked to identify Valentina’s or Flavia’s facial expression, by clicking on the matching schematic face below the photograph. In order to make sure that each child knew which emotion was depicted on the different schematic faces they were verbally labelled during the instruction video before starting the task. The schematic faces were identical to those used in Gao and Maurer’s studies [44, 50], and the order of presentation of the photographs and the relative positions of the schematic faces below each photograph were randomly set. Upon completion of the two tasks, all children were thanked for taking part in the games and debriefed in accordance with procedures established for Cyberball studies [56].

### Stimuli

The stimuli were extracted from Gao and Maurer’s [50] study and were composed of a total of 20 photographs posed by two women expressing either anger, fear or neutral expressions. Stimuli were selected from the NimStim Set of Facial Expressions ([57] models 3 and 10), based on adults’ high accuracy and intensity ratings for the expressions of anger (M_acc_ = 95%, M_int_ = 6.1 on a 7-point scale) and fear (M_acc_ = 81.5%, M_int_ = 5.9 on a 7-point scale) [57, 58]. They were coloured photographs with a resolution of 506 x 650 pixels. Contrary to most studies using only peak intensities, we chose to display photographs of emotional expressions at varying levels of intensity. Indeed, recognizing subtle expressions of emotions might be a great asset for successful social interactions, particularly when attempting to reconnect with others after being excluded. In addition, in the eventuality of children performing perfectly at the highest intensities of emotion, differences in emotion recognition abilities might be visible only at lower intensities. Thus, we decided to use Gao and Maurer’s stimuli [44, 50], who morphed photographs (selected from the NimStim database; [57]) of highly intense facial emotional expressions with photographs of neutral faces of the same models to create different levels of intensity. In this way, they created 20 levels of intensity with 5% increments, ranging from 5 to 100% (for details, see [48]). After Bayet and colleagues [59] we decided to present only two woman’s faces to children, and since our study was held online and comprised two different phases (i.e., Cyberball game and facial recognition task), we kept only a subset of these stimuli by reducing the number of intensity levels to 4 (i.e., intensity of 25%, 50%, 75% and 100%). Thus, each model expressed two different emotions at four levels of intensities as well as two neutral faces, resulting in a total of 20 photographs (2 models x 2 emotions x 4 intensities, plus 2 models x 2 neutral faces). As in Gao and colleagues [44, 50], the neutral expressions prevented children from thinking that all faces necessarily expressed a negative emotion, and were used for the calculation of two of the variables described in the analysis part below (i.e., threshold and misidentification).

### Analysis

Statistical analyses were performed on three different dependent variables. First, similarly to most emotion recognition studies [12, 16, 22, 60], we calculated the mean accuracy for each emotion (i.e., fear and anger), that is, the proportion of correct identifications of the emotion over all its different intensities. Following Gao and Maurer [44, 50], two other dependent variables were also examined. Indeed, having various levels of intensities meant that children could make two different types of errors. The first type of error involded a failure to detect any emotion on a low-intensity emotional face, mistaking it for a neutral face. It was measured by calculating the thresholds at which children detected an emotion on a face, identifying it as non-neutral. The second type of error was the misidentification of an emotion (i.e., mistaking one emotion for another). This type of error was calculated by measuring the percentage of misidentification for all faces that were recognized as non-neutral (i.e., above the threshold). These two latter measures provide complementary and more precise information concerning children’s ability to recognize the observed emotional expressions. Indeed, differently from the threshold and misidentification measures, the analysis of accuracy confounds the two types of mentioned errors: for example, seeing an angry face as neutral and mistaking a fearful face as angry. Thus, the threshold score provides a more nuanced comprehension of whether the observed intensity of a facial expression is sufficient to distinguish it from a neutral expression, while misidentification offers a more precise measure of errors resulting from assigning a facial expression to an incorrect emotion category.

#### Accuracy

The mean accuracy was calculated for each emotion by summing the number of correct identifications of that emotion over the different intensities (i.e., at 25%, 50%, 75% and 100% intensity) and dividing it by the total number of intensities (i.e., 4). Accuracies were calculated for each participant by averaging the accuracies obtained across the two models for each emotional expression (i.e., fear and anger).

#### Threshold

The threshold represented the level of intensity at which children identified an expression as non-neutral, that is, children’s sensitivity to this emotion. To calculate it, responses were divided into two categories: the neutral responses, when children did not detect any emotion at all (e.g., fear identified as neutral), and the non-neutral responses, when children detected an emotion, whether it was correct (e.g., fear identified as fear) or mistaken (e.g., fear identified or anger). After Gao and Maurer [44, 50], the responses of each participant to each emotion were then fitted with a cumulative Gaussian function to obtain a probability of identification of 0.5. That is, the threshold represented the intensity at which 50% of the time children identified the emotional face as neutral, and 50% of the time they identified it as expressive. The thresholds were calculated for each participant by averaging the thresholds obtained across the two models for each expression. Thus, a low threshold meant that the child successfully identified a face expressing an emotion at a low level of intensity as non-neutral. In sum, the child showed a high sensitivity to this emotion.

#### Misidentification rate

The misidentification rates represented the frequency of erroneous identifications of emotions among the faces that were recognized as non-neutral. Considering only the data above the threshold, it was calculated by dividing the frequency of misidentification by the total number of responses above the threshold. The misidentification rates were calculated for each participant by averaging the rates obtained across the two models for each expression.

### Statistical analysis

The same statistical analysis was performed for accuracies, thresholds and misidentification rates. Since preliminary analysis indicated no effect of the model on the results, all analyses were performed on values averaged across both models. In order to investigate the effect of ostracism on emotion recognition in the two age groups, we conducted a linear mixed model analysis, with age group (5- and 10-year-olds), condition (*inclusion* and *ostracism*), and emotion (anger and fear) as fixed effects, and intercept as a random effect. Furthermore, to explore the eventual interaction effect between age, condition and emotion factors, linear mixed model analyses will be performed in each age group independently, with condition (*inclusion*, *ostracism*) and emotion (anger and fear) as fixed effects, and intercept as a random effect. For each age group with a significant main effect or interaction, planned comparisons will be conducted using independent samples t-tests to examine the effect of the condition for each emotion separately, and paired sample t-tests to investigate differences in recognition of the different emotions within each condition. All these comparisons were planned a priori, based on our hypothesis that ostracism may differently affect the recognition of the two emotions.

Pairwise comparisons were performed by applying t-tests and Fisher’s least significant difference procedure [61], and Holm–Bonferroni correction was used where appropriate [62]. After Nakagawa & Schielzeth [63] effect sizes for mixed models were computed as the difference in R^2^ between the full-factorial and the basic model (i.e., not including the selected effect of interest). In addition, effect sizes for t-tests were estimated using Cohen’s *d* measure, and the data are reported as means and standard deviations (SDs). All statistical analyses were performed on Jamovi 1.6.15 (https://jamovi.org) using a two-tailed .05 level of significance.

## Results

### Accuracy

The linear mixed model performed on accuracies revealed a significant main effect of age group, *F*(1, 96) = 13.13, *p* < .001, with 5-year-olds showing lower accuracy than 10-year-olds (5-year-olds: *M* = 0.76, *SD* = 0.13; 10-year-olds: *M* = 0.82, *SD* = 0.12). In addition, a significant main effect of emotion was found, *F*(1, 96) = 26.70, *p* < .001, with children identifying more accurately expressions of fear (*M* = 0.83 *SD* = 0.14) than anger (*M* = 0.75, *SD* = 0.11). In sum, 10-year-olds were overall more accurate at recognizing emotions than 5-year-olds, and all children were overall more accurate at recognizing fear than anger. Importantly, these main effects were qualified by a significant emotion x age group x condition interaction, *F*(1, 96) = 4.41, *p* = .04, which was inspected by means of two separate 2 (condition) x 2 (emotion) linear mixed model analyses for each age group. The *ΔR*^2^ for the model was .038.

#### 5-year-old children

**Fig 1:**
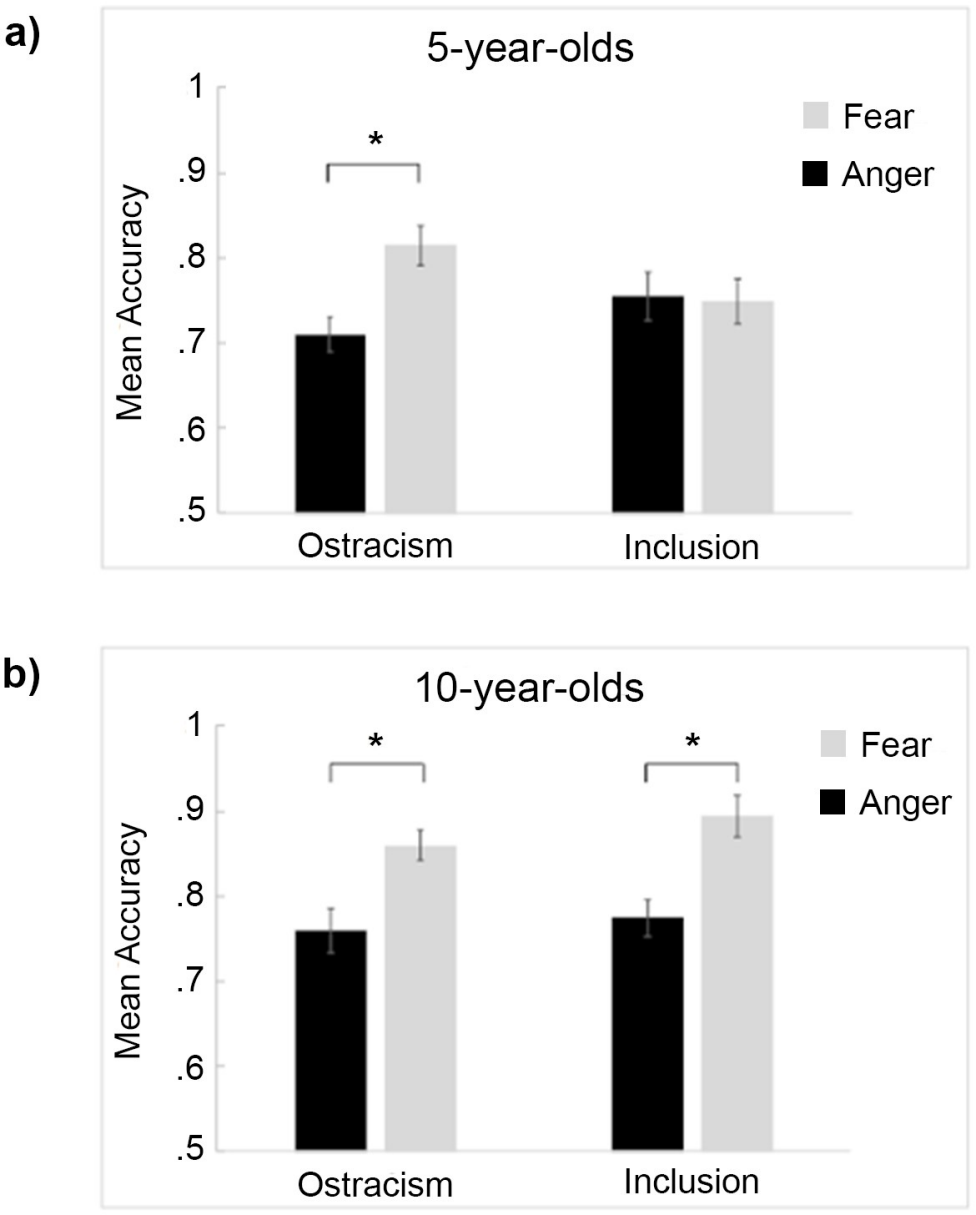
Mean accuracy scores (±SE) in response to angry (black) and fearful (light grey) expressions for 5-year-old (a) and 10-year-old children (b) in the inclusion and ostracism conditions. * indicates significant differences (*p* < .05).

The linear mixed model performed on 5-year-olds’ accuracy revealed a significant main effect of emotion, *F*(1, 48) = 4.90, *p* = .03, with fear being identified more accurately (*M* = 0.78, *SD* = 0.14) than anger (*M* = 0.73, *SD* = 0.11). This main effect was qualified by a significant interaction between emotion and condition, *F*(1, 48) = 5.93, *p* = .02. Planned comparisons revealed that ostracized children identified more accurately fearful (*M* = 0.81, *SD* = 0.13) than angry expressions (*M* = 0.71, *SD* = 0.11; Fig 1a), *t*(24) = 3.20, *p* = .02, d = .64, Conversely, no difference was observed in the inclusion condition, and the comparisons between the two conditions for each emotion separately did not reach statistical significance (all *p*s > .30). The *ΔR*^2^ for the model was .045.

#### 10-year-old children

The linear mixed model conducted on 10-year-olds’ accuracy revealed a significant main effect of emotion, *F*(1, 48) = 26.95, *p* < .01, with fear being identified more accurately (*M* = 0.88, *SD* = 0.11) than anger (*M* = 0.77, *SD* = 0.11; Fig 1b). No significant main effect of condition nor any interaction was observed (all *p*s > .30). Planned comparisons were nonetheless conducted to further confirm that both included and ostracized children identified more accurately fearful than angry expressions (inclusion: fearful *M* = 0.89, *SD* = 0.11; angry *M* = 0.77, *SD* = 0.13, *t*(24) = 3.67, *p* = .004, d = .73; ostracism: fearful *M* = 0.86, *SD* = 0.12; angry *M* = 0.76, *SD* = 0.09 *t*(24) = 3.70, *p* = .04, d = .74). The *ΔR*^2^ for the model was .193.

### Threshold

The linear mixed model performed on threshold scores revealed a significant main effect of age group, *F*(1,96) = 12.36, *p* < .001, with 5-year-olds showing higher thresholds (*M* = 37.00%, *SD* = 12.90%) than 10-year-olds (*M* = 30.50%, *SD* = 12.70%). A significant main effect of emotion was also observed, *F*(1,96) = 25.97, *p* < .001, with thresholds for anger (*M* = 37.70%, *SD* = 11.30%) being higher than thresholds for fear (*M* = 29.80%, *SD* = 13.70%). In sum, 5-year-old children were less sensitive to subtle expressions of emotions than 10-year-olds, and children were overall less sensitive to low-intensity expressions of anger as compared to fear. Finally, a marginal interaction was observed between the age group, emotion and condition, *F*(1,96) = 3.86, *p =* .05, which was further explored by means of two separate 2 (condition) by 2 (emotion) linear mixed model analyses for each age group. The *ΔR*^2^ for the model was .036.

#### 5-year-old children

**Fig 2:**
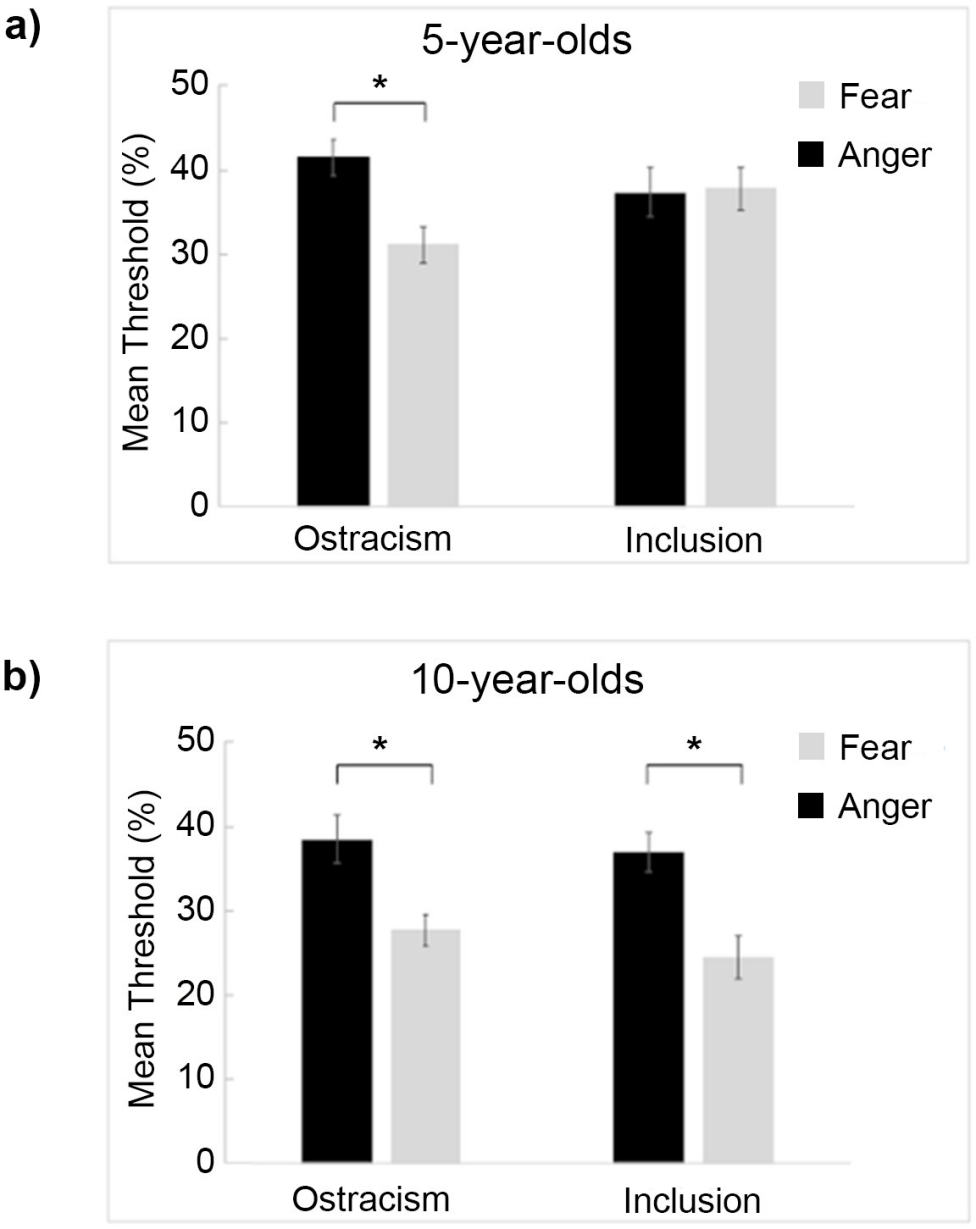
Mean threshold (±SE) for angry (black) and fearful (light grey) expressions for 5- (a) and 10-year-old children (b) in the inclusion and ostracism conditions. * indicates significant differences (*p* < .05).

The linear mixed model performed on 5-year-olds’ thresholds revealed a significant main effect of emotion, *F*(1,48) = 4.78, *p =* .03, with children showing higher thresholds for anger (*M* = 39.40%, *SD* = 11.10%) than fear (*M* = 34.50%, *SD* = 14.10%), which was qualified by a significant emotion by condition interaction, *F*(1,48) = 5.80, *p =* .02. Planned comparisons showed a significant difference in threshold between anger and fear in the ostracism condition, *t*(24) = 3.19, *p* = .02, d = .64, with ostracized children showing higher threshold for anger (*M* = 41.50%, *SD* = 10.80%) than for fear (*M* = 31.20%, *SD* = 13%; Fig 2a). No other comparison attained statistical significance (all ps > .30). No significant main effect of condition was observed (*p* = .66). In sum, 5-year-old children who were previously ostracized were less sensitive to subtle angry than to subtle fearful expressions. The *ΔR*^2^ for the model was .043.

#### 10-year-old children

The linear mixed model performed on 10-year-olds’ thresholds only revealed a significant main effect of emotion, *F*(1,48) = 25.62, *p* < .001, with children showing higher thresholds for anger (*M* = 36.00%, *SD* = 11.40%) than for fear (*M* = 25.00%, *SD* = 11.60%; Fig 2b). In sum, 10-year-olds were more sensitive to subtle expressions of fear than anger. No main effect or interaction involving condition was found (all *p*s > .36). Planned comparisons were nonetheless conducted to further confirm that both included and ostracized children exhibited lower thresholds for fearful than angry expressions (inclusion: fearful *M* = 23.50%, *SD* = 11.10%; angry *M* = 35.30%, *SD* = 13.40%; *t*(24) = 3.46, *d* = .69, *p* = .006; ostracism: fearful *M* = 26.50%, *SD* = 12.14%*;* angry *M* = 36.80%, *SD* = 9.03%*; t*(24) = 3.79, *p* < .004, d = .76). The *ΔR*^2^ for the model was .187.

### Misidentification rate

**Fig 3:**
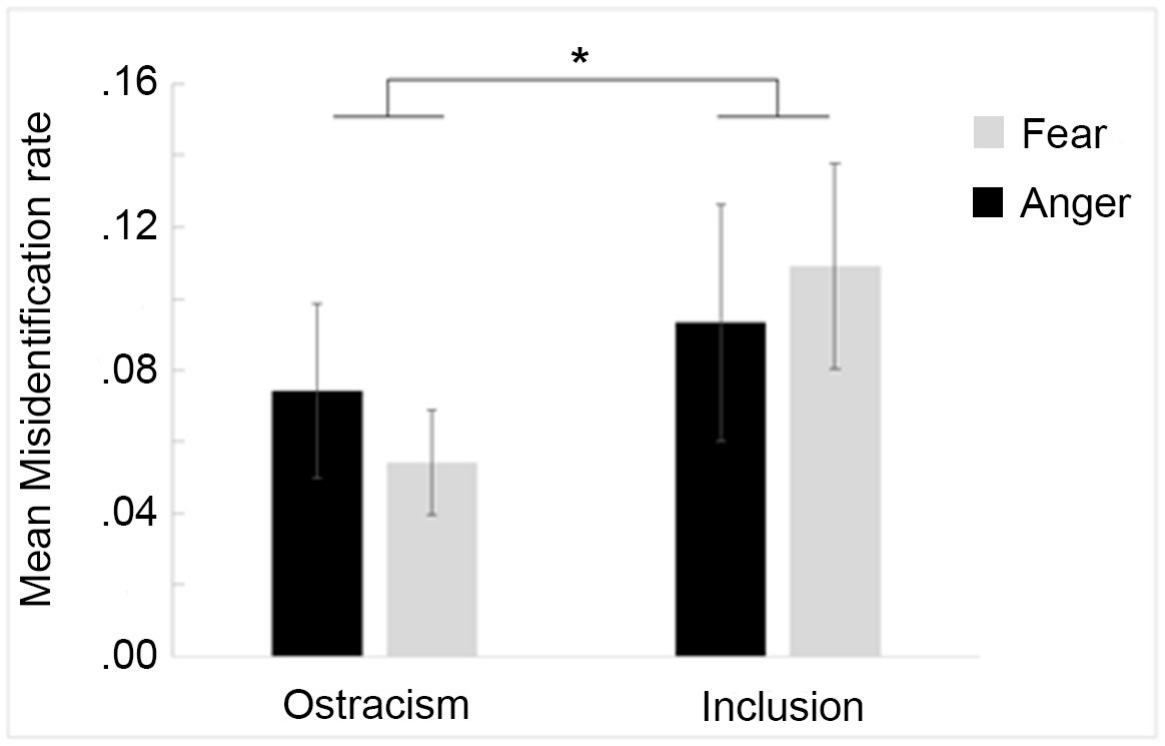
Mean misidentification rates (±SE) for angry (black) and fearful (light grey) expressions in the *ostracism* and *inclusion* conditions. * indicates a significant difference.

The linear mixed model performed on misidentification rates revealed a significant main effect of condition, *F*(1,192) = 4.07, *p* = .04. Indeed, children who were previously ostracized showed lower misidentification rates (*M* = 0.06, *SD* = 0.10) than included children (*M* = 0.10, *SD* = 0.15; Fig 3). No other significant main effect or interaction was observed (all *p*s > .11). The *ΔR*^2^ for the model was .019.

## Discussion

It is known that ostracism affects adults’ processing of social signals. Nonetheless, available literature examining the consequences of ostracism on children has never investigated its impact on children’s face processing and specifically on their capacity to recognize emotional faces.

The current study was aimed at investigating whether, in line with the social monitoring model [15], social manipulations of inclusion and ostracism modulate children’s ability to recognize facial expressions of emotions displayed at different levels of intensity. To do so, 5- and 10-year-old children participated in an online Cyberball game and then completed a facial emotion recognition task in which they were required to identify emotions (or the absence of emotion) on faces displaying either neutral expressions or varying levels of intensity of angry and fearful facial expressions (ranging from low, i.e., 25% level of intensity, to peak intensity). As expected, the two age groups differed in their overall accuracy and sensitivity to facial expressions, with 5-year-olds being less accurate and less sensitive to fearful and angry faces than 10-year-olds. This finding is in agreement with previous research showing that children’s accuracy at recognizing emotions gradually improves with age and undergoes a sharp increase between 3- and 7-year-olds [40–42]. This improvement in children’s performance might reflect increasing cognitive and socio-emotional abilities, such as attention, understanding of social and emotional situations, or perspective-taking [64, 65], which facilitates the recognition of emotional expressions in older children.

Consistent with the few studies showing that Cyberball-induced social manipulations can affect diverse aspects of children’s cognition [26, 30, 31], the current study showed that inclusion and ostracism also elicited significant changes in children’s recognition of emotional facial expressions. In particular, results indicated lower misidentification rates for children who were previously ostracized as compared to children who were previously included, independently of children’s age and emotion observed. Thus, misidentification results are consistent with prior findings in adults [12, 16], implying that ostracism enhanced children’s emotion decoding abilities. This aligns with Gardner and colleagues’ model [15], proposing that individuals possess a social monitoring system that directs attention and cognitive resources on social cues when their belonging needs are threatened. Thus, in an ostracism scenario, proficiently decoding others’ emotions could be a useful asset for facilitating re-inclusion.

A partially different result pattern was observed for accuracy and sensitivity (i.e., threshold) measures, as only 5-year-olds’ decoding abilities were affected by the social manipulation, while no difference between the *inclusion* and *ostracism* conditions was observed for 10-year-olds. Indeed, at age 10, all children showed higher accuracy and sensitivity for expressions of fear as compared to anger. Different interpretations may be proposed for the absence of a significant effect of the Cyberball manipulation on accuracy and sensitivity in 10-year-old children. One possibility is that the overall better performance of 10-year-olds may have masked the effect of our main experimental manipulation. Another possibility is that, with development, the evolutionary function of fear as a signal of potential danger in the environment becomes particularly relevant, regardless of the social context, leading to increased prioritization of recognizing fearful faces over other emotional expressions like anger. This interpretation aligns with prior work suggesting that facial emotion recognition continues to mature during childhood, with fear being one of the last emotions to be accurately identified [66]. Furthermore, it is plausible that the specific design employed in the current study may have hindered the observation of more subtle differences in children’s emotion recognition abilities following the Cyberball manipulation. The limitation of using only four intensity levels (i.e., 25%, 50%, 75%, and 100%) may have hindered the detection of finer differences in accuracy and sensitivity, including differences below 25% of intensity, as observed in Gao and Maurer [44]. Also, the use of an online procedure in our study (see below) may have reduced the effectiveness of the task for 10-year-olds. Further research investigating the impacts of social inclusion and ostracism on children’s processing of emotional expressions is necessary to provide a more definitive understanding of this issue.

Crucially, the Cyberball manipulation significantly impacted 5-year-olds’ emotion recognition abilities, considering accuracy and sensitivity measures. Indeed, ostracized 5-year-olds were more accurate and sensitive to fearful than angry expressions, while included 5-year-olds did not exhibit this difference. This suggests that, at 5 years of age, the prioritization of the decoding of fearful faces is maintained only in socially-threatening contexts such as ostracism, while a safe context of inclusion might constitute a social signal to stop prioritizing the decoding of fear over other expressions. Consistently, our findings revealed an advantage in processing fearful faces, except in the inclusion condition for younger children. These results suggest that the ability to accurately recognize fearful faces at an earlier developmental stage may be more permeable to the effects of positive or negative social interactions, such as being included or ostracized by other peers. Overall, our results are consistent with prior research showing the impact of ostracism on children’s cognitive abilities [26, 30, 31]. Indeed, we observed differences between inclusion and ostracism conditions in all three measures (i.e., accuracy, sensitivity, and misidentification rates) related to emotion recognition, although the extent of modulation varied across measures and age groups.

Obtained results for the misidentification rate were the same for both emotions and age groups, with ostracized children showing lower overall misidentification rates than included children. These findings are in line with data deriving from studies conducted with adults [12, 16] and with the social monitoring system model [15], according to which excluded individuals should exhibit a general improvement in emotion recognition (i.e., for all emotions). Interestingly, our results also suggest that inclusion might lead to a decrease in 5-year-olds’ accuracy and sensitivity specifically for fearful expressions. This modulation of recognition capacities was solely observed for fearful expressions, and only in the youngest age group. Thus, while adults’ emotion processing is mostly affected by the manipulation of ostracism rather than inclusion [12, 16], our results indicate that the higher recognition performance recorded for fearful stimuli was consistently present, but absent in the younger age group when socially included. This suggests that younger children appear particularly affected by social inclusion rather than ostracism.

Despite this study used a robust and widely employed experimental paradigm for ostracism, there are some limitations that need to be addressed in future research. Indeed, available literature in social psychology suggests that inclusion serves as a suitable control condition for ostracism [66], as it implies an expected and non-elevatory level of inclusion. However, upcoming studies could examine whether incorporating an additional control condition (e.g., Cybertree), might reveal different results concerning the impact of ostracism on children’s capacity to recognize facial emotional expressions. Importantly, while the use of an online procedure might represent a further element of originality and novelty of the current study, it might as well imply possible limitations. Indeed, online experimental studies intrinsically provide a series of advantages well documented in recent literature, such as (1) easy access to a demographically and culturally diverse participant population; (2) bringing the experiment to the participant instead of the opposite; (3) high statistical power due to the possibility to involve large samples; and (4) cost savings of lab space, person-hours, equipment, and administration [67–69]. Nonetheless, the fact that our study was held online might have entailed some differences in testing conditions across participants (e.g., size of the screen, familiarity with the use of the computer, environmental distractions), as well as a lack of control of these conditions, that might have masked or amplified potential effects, thus modulating children’s performance. In addition, the online participation also implied that the experiment took place in the children’s homes, in the presence of parents and several possible distractions. As no catch trials were included in the current procedure, this raises the important issue of the need to control participants’ attention, especially in online procedures, as the current procedure was not designed to evaluate participants’ attention during the task. Thus, future studies are required to further investigate this aspect and provide more confidence in the obtained results. Additionally, as children were tested at home in the presence of parents, it is possible that they may have affected children’s performance. Precise instructions were given to parents, who were asked to sit back, never help the child, and limit their intervention to strictly defined situations in which the child did not intend to continue the task. Yet, the closeness of a parent by itself might have somehow modulated children’s reactions to inclusion or ostracism during the Cyberball paradigm as well as children’s performance on the recognition of emotional faces. Future studies in which an experimenter can monitor the behaviour of the child in person and in the absence of the possible interference of parents will clarify these aspects. Note however that several studies recently investigated children’s face processing using an online procedure [e.g., 70] and that we applied the same online procedure to all conditions and participants, thus likely limiting the impact of any intervening variable.

Furthermore, although the Primary Needs Questionnaire–Children [71], which could reliably assess participants’ reaction to the Cyberball manipulation, is not available for children under 6 years of age, our online procedure allowed parents to report a brief written note about their children’s reactions to the experimental session. Indeed, parents of children assigned to the ostracism condition frequently (35% of 5-year-old children; 87% of 10-year-old children) reported that their child manifested or verbalized feelings of distress and sadness, while no signals of sadness or distress were noted by the parents of included participants. It would be interesting for future research to examine this aspect further by using more implicit measures, such as coding of children’s behavioural reactivity during the Cyberball or using children’s affiliative drawings [27, 72].

To sum up, the current study presented evidence that Cyberball-induced social manipulations modulate children’s recognition of facial expressions, constituting another step towards a better comprehension of the extensive range of effects brought by social inclusion and ostracism. To fully examine the effects of social inclusion and ostracism across development, future research should investigate its physiological and psychological consequences using the same experimental design and methodology at different developmental stages. This could allow us to better understand the developmental trajectories of the strategies adopted to cope with ostracism.
